# Current Advances in Pharmacotherapy and Technology for Diabetic Retinopathy: A Systematic Review

**DOI:** 10.1155/2018/1694187

**Published:** 2018-01-17

**Authors:** Lei Lu, Ying Jiang, Ravindran Jaganathan, Yanli Hao

**Affiliations:** ^1^Department of Ophthalmology, Yan'an People's Hospital, Qilipu St., Baota Qu, Yan'an, Shaanxi 716000, China; ^2^Pathology Unit, Lab Based Department, Faculty of Medicine, Royal College of Medicine Perak, Universiti Kuala Lumpur, No. 3, Jalan Greentown, 30450 Ipoh, Perak, Darul Ridzuan, Malaysia; ^3^Department of Ophthalmology, Yan'an People's Hospital, Bei Da Jie, Baota Qu, Yan'an, Shaanxi 716000, China

## Abstract

Diabetic retinopathy (DR) is classically defined by its vascular lesions and damage in the neurons of the retina. The cellular and clinical elements of DR have many features of chronic inflammation. Understanding the individual cell-specific inflammatory changes in the retina may lead to novel therapeutic approaches to prevent vision loss. The systematic use of available pharmacotherapy has been reported as a useful adjunct tool to laser photocoagulation, a gold standard therapy for DR. Direct injections or intravitreal anti-inflammatory and antiangiogenesis agents are widely used pharmacotherapy to effectively treat DR and diabetic macular edema (DME). However, their effectiveness is short term, and the delivery system is often associated with adverse effects, such as cataract and increased intraocular pressure. Further, systemic agents (particularly hypoglycemic, hypolipidemic, and antihypertensive agents) and plants-based drugs have also provided promising treatment in the progression of DR. Recently, advancements in pluripotent stem cells technology enable restoration of retinal functionalities after transplantation of these cells into animals with retinal degeneration. This review paper summarizes the developments in the current and potential pharmacotherapy and therapeutic technology of DR. Literature search was done on online databases, PubMed, Google Scholar, clinitrials.gov, and browsing through individual ophthalmology journals and leading pharmaceutical company websites.

## 1. Introduction

Diabetic Mellitus (DM), a chronic metabolic disorder, is a major public health problem due to its associated complications [1]. One of the major complications of DM is DR, which is an important cause of preventable blindness. In the United States, DR is the leading cause of blindness for patients with age of 20 to 64 and accounts for about 12% of all new cases [2]. Moreover, 80% of patients with DM for more than 20 years are most commonly diagnosed with DR. Nonetheless, at least 90% of new cases were shown to have retinal structure and function restoration when proper treatments were applied [3].

DR is characterized by the progressive damage in the retinal microvasculature. It can be classified into nonproliferative DR (NPDR) and proliferation DR (PDR) [4, 5]. NPDR is featured with intraretinal microvasculature changes [6] and can be further divided into mild, moderate, and severe stages that may associate with diabetic macular edema (DME) [7]. PDR involves the formation and growth of new blood vessels (retinal neovascularization) in low oxygen condition [6]. Thus, the newly formed blood vessels are fragile and without timely treatment may lead to vision loss. In most cases, DR is also featured with increased vascular permeability, leading to fluid accumulation and retinal hemorrhages in the macula, all of which referred to DME [8–10].

There are three major treatments of DR, such as laser surgery, pharmacotherapy, and vitrectomy, which are effective to reduce vision impairment [11]. Intraocular pharmacotherapy, particularly applying anti-inflammatory (corticosteroids) and anti-angiogenic agents (VEGF inhibitors), has become the most preferred therapy for DR [12]. The advantage of intraocular pharmacotherapy is that multiple intraocular injections decrease the treatment burden of some patients, but others with chronic DME may require intensive longer-term therapy. Recent studies showing that intravitreal corticosteroid and anti-VEGF agents have promising results in treating the progression of PDR and DME, which lead to increased use of these agents. However, its short-duration effects and severe side effects limit its use [13]. Indirectly, these agents are unable to substitute the panretinal photocoagulation, which provides longer effects and effectiveness in preventing vision loss in the late stages of DR. Therefore, pharmacotherapy is still considered an adjunct treatment to panretinal photocoagulation. Further searching for a better pharmacotherapy has led to the discovery of recently approved anti-VEGF drugs (aflibercept) and corticosteroids (dexamethasone and fluocinolone inserts) [14, 15]. These drugs provide longer durations of action. Further, danazol and minocycline (oral medications) and loteprednol (modified topical drugs) require daily administration, thus decreasing the necessity of patients to visit physicians [12]. In addition, drugs administered using the intravitreal route as either monotherapy or combination therapy in targeting different biochemical pathways that induce DR are also developed. However, their durability and effectiveness need further monitoring [12]. Therefore, longer action drugs, sustained drug delivery systems of corticosteroids and anti-VEGF, and encapsulated cell technology may further decrease treatment burden, though regulatory approval may not occur for at least 5 years.

## 2. Methodology

The literature search was conducted on PubMed with no limitation on language or year of publication. The review focuses on the clinically used drugs/proteins along with a brief background on the path physiology of DR. The major focus of this review revolves around current and new drugs/proteins, drug delivery system, drug delivery devices, and potential encapsulated cell technology in treating DR. In each category, major advances are discussed along with the possible solutions.

## 3. Pathophysiology

### 3.1. Growth Hormone

The exact mechanism of how DM causes DR is unclear. However, several theories have been postulated to explain the typical course and history of DR [16]. One of the theories is growth hormone. Growth hormone has been demonstrated to affect the development and progression of DR. Women diagnosed with hemorrhagic necrosis of the pituitary gland, called Sheehan syndrome, are associated with a reversible DR [17]. In 1950, pituitary ablation was used to treat or prevent DR; however, this practice is considered controversial and has been abandoned due to many systemic complications and the discovery of laser surgery [17]. Besides, patients with hypopituitarism were diagnosed with DR [17].

### 3.2. Platelets and Blood Viscosity

DM patients are frequently associated with retinal ischemia, which is a risk factor to contribute to the development of DR. DR patients with retinal ischemia are found to have various hematologic abnormalities, including increased red blood cell aggregation, decreased red blood cell deformability, increased platelet aggregation, and adhesion. Further, the patients are prone to have sluggish circulation, endothelial damage, and focal capillary occlusion [18].

### 3.3. Aldose Reductase (AR) and Vasoproliferative Factors

DM is caused by the abnormality in the glucose metabolism, mostly due to the decreased levels or activity of insulin. Both structural and physiologic effects are induced by increased levels of glucose, causing the retinal capillary to be functionally and anatomically abnormal [19]. The effects of excess glucose on retinal capillary are due to the biochemical changes in the polyol (or aldose reductase (AR)) pathway [20]. In the polyol pathway, glucose is metabolized into sorbitol. Accumulation of sorbitol results in intramural pericytes of retinal capillaries losing their primary function, for example, auto regulation of retinal capillaries [20]. Eventually, this lead to weakness and saccular outpouching of the capillary walls (or termed microaneurysms). Microaneurysms are the earliest detectable symptoms for DR. Ruptured microaneurysms cause retinal hemorrhage. Subsequently, increased permeability of the vessels leads to retinal thickening due to leakage of fluids and proteinaceous substances [18]. A diminution in central vision can be involved in retinal thickening happens in the macula [20]. Nailfold video capillaroscopy can be used to examine the retinal damage-related capillary changes, indicating a generalized microvessel involvement in both type 1 and 2 diabetic patients [18, 21].

### 3.4. Macular Edema

Macular edema is one of the most common of vision loss. It is usually detected in patients with NDPR, and it may also complicate the patients with PDR. The development of macular edema is caused by the increased levels of diacylglycerol during the shunting of excess glucose. Increased levels of diacylglycerol activate the protein kinase C, leading to fluid leakage and retinal thickening [22, 23].

### 3.5. Hypoxia

As the disease progresses, the closure of the retinal capillaries induces hypoxia (low oxygen perfusion). Further, infarction of the nerve fiber layer forms cotton-wool spots, with stasis in axoplasmic flow association [20]. Various compensatory mechanisms are activated by extensive hypoxia to provide sufficient oxygen to tissues. Increasing hypoxia causes venous caliber abnormalities, such as venous beading, loops, and ablation, which can be observed in the areas of capillary nonperfusion. This observation subsequently leads to intraretinal microvascular abnormalities, featured with the growth of new vessels and remodeling of preexisting vessels via endothelial cell proliferation [20].

### 3.6. Neovascularisation

Persistent retinal ischemia results in the production of vasoproliferative factors to stimulate new vessel formation. Firstly, the extracellular matrix of the retina is first broken down by the proteases followed by growth of new vessels from the retinal venules and penetrates the internal limiting membrane and form capillary network between the inner surface of the retina and the posterior hyaloid face [20]. Nocturnal intermittent hypoxia, a risk factor for iris and/or angle neovascularization, arises from sleep-disordered breathing in patients with PDR [24]. Neovascularization can be found at the borders of perfused and nonperfused retina and most commonly along with the vascular arcades and at the optic nerve head [20]. The newly formed vessels are fragile and easily disrupted by vitreous traction, resulting in hemorrhage in the vitreous cavity or preretinal space [20]. In addition, these new vessels are initially associated with a small amount of fibroglial tissue formation, which increases as the density of the neovascular frond increases [16]. Gradually, the vessels may regress and only the networks of avascular fibrous and tissue can be adherent on the retinal and posterior hyaloid face. The tractional forces applied on the retina via these fibroglial connections, may cause retinal edema, retinal heterotropia, and retinal detachment and retinal tear formation with subsequent detachment [20].

## 4. Therapeutic Approaches and Their Current Delivery System

The pharmacotherapy for the treatment of DR is mainly resolved around corticosteroid therapy and anti-VEGF agents. Besides, the potential plant-based drugs, vitreolytic agents, systemic agents such as hypoglycemic, hypolipidemic, and antihypertensive, and pluripotent stem cells technology in treating the progression of DR are also discussed. The background of the delivery system for these existing or newly discovered drugs is also briefly discussed.

### 4.1. Anti-Inflammatory Agents

Corticosteroids have been reported to possess high anti-inflammatory and antiangiogenesis activities [13] by modulating the various proinflammatory mediators, such as tumor necrosis factor-alpha (TNF-*α*), interleukin-1*β* (IL-1*β*), and VEGF. These mediators are upregulated during DR progression [13]. The primary design for delivering corticosteroids is via direction injection (intravitreal route) in order to avoid the blood-retinal barrier limitations to reach the target site. Corticosteroid therapy has been widely used as a treatment option for DME and DR; however, their use is limited due to the short-duration effects. Moreover, direct intravitreal injections were reported to have severe side effects, such as cataract and elevation of IOP [25]. Other less common side effects of these injections include retinal detachment, vitreous hemorrhage, and endophthalmitis [26, 27]. Nonetheless, the systemic use of corticosteroid therapy can be an effective adjunct to laser photocoagulation to treat DME and DR, as well as improve the condition of best-corrected visual acuity (BCVA) [28].

Intravitreal triamcinolone acetonide (IVTA) has been reported to have potent anti-inflammatory activity and improve the condition of DME and age-related macular degeneration (AMD) [29–33]. The effect of IVTA is transient and possibly last for three months. Thus, multiple injections of IVTA are required in order to maintain its effectiveness. IVTA is also capable of reducing PDR due to its potent antiangiogenic effects [34]. Thus far, the United States Food and Drug Administration (FDA) has approved two IVTA preservative-free injections with a recommended dose usage, including triesence (40 mg/ml, Alcon) and trivaris (80 mg/ml, Allergan) to alleviate the condition of noninfectious endophthalmitis and other complications [34]. The effectiveness of lower doses of trivaris (1 and 4 mg) was tested by the DR clinical research (DRCR) network in a large, multicenter randomized clinical trial for the treatment of macular edema. In comparison to trivaris, treatment of macular photocoagulation showed improved BCVA and fewer complications [13]. However, trivaris treatment caused a lower rate of endophthalmitis (0.05%) in patients enrolled throughout the three years of follow-up [35]. Next, trivaris administered at three months demonstrated improved BCVA, but no effects at a longer period of treatment as suggested by many randomized clinical trials [36]. In this regard, the development of novel IVTA delivery devices that are released in a small quantity in a sustained system is required.

### 4.2. Corticosteroid Therapy with Sustained Delivery System

Sustained-release corticosteroid therapy has been developed to treat chronic DR and also reduced the associated adverse effects. One of such delivery systems for use in DME is triamcinolone acetonide (TA) implant (I-vation®). I-vation is currently completed in phase I clinical trial for the long-term treatment of DME while the second clinical trial of TA implant is still under planning [37, 38]. This drug delivery device is made of biodegradable polymers which degrade slowly for a period of time and bypass the risk of secondary surgical complications upon removal compared to nonbiodegradable devices [37].

Another sustained drug delivery system is fluocinolone acetonide nonbiodegradable intravitreal insert (Iluvien®, Alimera Sciences). Iluvien is designed to release fluocinolone for up to three years. Interestingly, this device is very small, which allows direct injection to the back of the eye with a self-sealing hole. It has been reported by a FAME study that low dose of Iluvien inserted (0.19 mg per day) showed improved BCVA compared to higher dose [14, 15, 39]. Currently, Iluvien is still under investigation by FDA to approve its application to the market [13]. In comparison, another fluocinolone acetonide intravitreal implant, Retisert® (Bausch and Lomb), is approved by FDA for the treatment of chronic, noninfectious uveitis. However, its treatment caused severe cataract and glaucoma in a phase III clinical trial [14, 15].

Besides, FDA has also approved Ozurdex (allergen), which is a sustained release of biodegradable dexamethasone intravitreal implant for the treatment of macular edema. A study that carried out in 2007 discovered that higher dose of dexamethasone (700 *μ*g) statistically improved that BCVA and well-tolerated by patients at three months compared to low dose (350 *μ*g). However, there was no further improvement in BCVA at six months and both doses caused IOP elevation [40, 41].

Next, Cortiject implant (NOVA63035; Novagali Pharma) is a tissue-activated proprietary corticosteroid prodrug that available in a preservative- and solvent-free emulsion. A single injection of the emulsion provides the efficacy for about six to nine months period. At the retina level, the prodrug is converted into active drug format. Currently, NOVA63035 is under phase I clinical trial with different settings of open-label and dose-escalation to test its safety and tolerability in patients diagnosed with DME [42].

Lastly, Verisome delivery system is also used for sustain-release of corticosteroids. This device is biodegradable and provides a controlled, extended release of drug in a titratable period for up to one year. Once released via a standard 30-gauge needle, the liquid coalesces into s spherule in the vitreous. Verisome technology is currently used in the clinical trials for the sustained-release delivery of TA (IBI-20089) [13, 43]. A single intravitreal injection of Verisome-delivered IBI-20089 was tested in phase I and II clinical trials for patients with cystoids macular edema associated with retinal vein occlusions (RVO) using two doses, with 25 *μ*l dose designed for six months period and 50 *μ*l dose designed for one year [44]. The 25 *μ*l dose was incorporated with a lower dose of triamcinolone (6.9 mg) while 50 *μ*l doses administered with a higher dose of triamcinolone (13.8 mg) [44]. The results showed that the patients were well-tolerated with Verisome without any related adverse effects. The lower dose of triamcinolone showed evidence of controlled release efficacy, with greater control was demonstrated with higher dose [44].

### 4.3. Other Nonsteroidal Anti-Inflammatory Agents

There are other nonsteroidal anti-inflammatory agents that have been approved by FDA to treat DR and DME. One of them is nepafenac (Nevanac®, Alcon), which is a topical nonsteroidal drug that has shown great efficacy to treat DME [45]. However, nepafenac testing in clinical trials is under way. Next, etanercept (Enbrel®, Amgen Inc., and Wyeth) is a recombinant fusion protein that targets TNF-*α*. It is approved by FDA to treat psoriasis [46]. It has shown that patients with refractory DME treated with intravitreal etanercept did not show any improvements [47]. Another, TNF-*α* antagonist is infliximab (Remicade®, Centocor) and it is used to treat Crohn's disease. Systemic treatment of DME with intravitreal infliximab injection achieved anatomic and functional improvement was achieved [48].

### 4.4. Antiangiogenic Agents

Antiangiogenic agents have been shown to effectively treat PDR and DME in addition to corticosteroid therapy. More novel and specific anti-angiogenic agents were discovered to solve the DME-associated vascular leakage and neovascularization. These antiangiogenic agent's primary targets the subfamily protein, VEGF, in which its over-expression plays a crucial role in the progression of DR and AMD [13]. Nonetheless, laser photocoagulation is still considered gold standard therapy for PDR; with anti-VEGF agents used as its adjunct treatment to reduce the adverse effects.

Bevacizumab (Avastin®, Genentech) is a full-length humanized antibody that targets all subtypes of VEGF [49]. Intravitreal injection of Avastin has led to the reduction of vascular permeability, and retinal and choroidal neovascularisation, all of which are precursor lesions for developing PDR and AMD [50–53]. In addition, intravitreal Avastin therapy was reported to reduce iris and retinal neovascularization [54]. However, the effect is short as recurrence of neovascularization was found after two weeks [54]. After all, Avastin is now widely used as a clinical adjunct to laser photocoagulation to treat patients with PDR [55, 56]. In 2006, FDA has approved ranibizumab (Lucentis®, Genentech Inc.), which is a recombinant humanized antibody fragment used to target VEGF-A to treat exudative AMD [57, 58]. In 2010, Lucentis was approved by FDA for the treatment of ME following RVO after evaluating its safety and efficacy profiles by BRAVO and CRUISE studies. In comparison with patients receiving sham injections, the patients treated with Lucentis met the primary endpoint of mean change from the baseline in BCVA at six months. The patients in the BRAVO study treated with Lucentis had a mean gain of 18.3 letters compared to 7.3 letters in the control patients at six months. A similar result pattern was observed in the CRUISE study, in which treated patients had a mean gain of 14.9 letters while control patients had 0.8 letters [59, 60]. Moreover, Lucentis was found to meet the primary endpoint of one of the two-phase III clinical trials (called RISE study) in January 2011 [13].

Another recombinant protein is VEGF trap-eye (aflibercept, EYLEA®, Regeneron Pharmaceuticals Inc., and Bayer HealthCare Pharmaceuticals) which fuse both human VEGF receptors 1 and 2 extracellular domains and a Fc portion of human IgG1 that binds all subtypes of VEGF-A along with the related placental growth factor. It has been tested in VIEW1 and VIEW2 clinical trials that patients were found to be well-tolerated with intravitreal VEGF trap-eye injection one month after Lucentis therapy [61, 62]. VEGF trap-eye is currently under marketing approval by European Medicines Agency for the treatment of wet macular degeneration [13].

JSM6427, an antiangiogenic compound developed by German biopharmaceutical company (Jerini AG) has been shown positive results in reducing DR. Sustained release of JSM6427 by using an intraocular implanted osmotic pump for an extended period of six months. Currently, JSM6427 is under phase I clinical trial [63].

TG100801 is a topical prodrug that may be effective in treating retinal disorders and it can be contemporary used with other approved products. It has been shown that topical administration of TG10001 alleviated the retinal changes, such as retinal leakage, angiogenesis, and inflammation that mediated by VEGF [64]. Once released, it converts to active drug, TG100572, which was reported to treat DR and wet macular degeneration by overcoming neovascularization and inflammation [65].

ATG3, a topical drop developed by CoMentis Inc. and designed to penetrate into retina and choroid effectively. ATG3 targets angiogenesis-mediated nicotinic acetylcholine receptor pathway. It is currently under phase III clinical trial for its use to treat neovascular AMD. Well-tolerability and no systemic sides effects were observed in 80 healthy controls treated with single- and multiple-dose ascending regimens for up to 14 days in a randomized, double-masked, placebo-controlled phase I study [65].

Lastly, an orally administered antiangiogenic drug, pazopanib was developed by GlaxoSmithKline. This drug is designed to target VEGF receptor (VEGFR), platelet-derived growth factor receptor (PDGFR), and c-kit. Currently, it is under phase III clinical trials to test its safety, efficacy, and well-tolerability [66, 67].

### 4.5. Vitreous Agents

Vitrase (hyaluronidase ovine, ISTA Pharmaceuticals Inc.) is the first and only pure, preservative-free, thimerosal-free, ovine hyaluronidase. FDA has approved its use as a spreading agent. It is currently in a phase III clinical study to test its capability in clearing vitreous hemorrhage from PDR [68].

Microplasmin is another vitreous agent and administered via intravitreal injection. It has been reported to treat DME and PDR by inducing posterior vitreous detachment. For example, ThromboGenics NV (Belgium) has carried out a randomized, double-masked, sham-injection controlled, dose-ascending clinical trial to compare the effects of multiple doses of intravitreal microplasmin for treatment of patients with DME compared to control patients. The finding showed that microplasmin has the ability to induce vitreous detachment by greatly reducing retinal neovascularization [69, 70]. Moreover, microplasmin was reported to cure focal vitreomacular adhesion as tested in two MIVI-TRUST phase III clinical trials. The trials demonstrated that microplasmin can resolve vitreomacular adhesion and cure full thickness macular hole and vision impairments and that it is safe and well-tolerated [71, 72].

### 4.6. The Potential Use of Systemic Agents to Treat DR

Many systemic agents that are primarily used to treat dyslipidemia and hypertension in diabetic patients were found to reduce the progression of DR. However, their mechanisms of action in treating DR need further investigations preclinically and clinically.

### 4.7. Hypoglycemic Agents

#### 4.7.1. Insulin Therapy

Insulin is used to primarily inhibit the progression of long-term diabetic complications. Many studies have reported that a good glycemic control is an effective treatment option to delay DR progression [73]. For example, Diabetes Control and Complications Trial (DCCT) demonstrated that intensive insulin therapy can induce retinal proangiogenic effects while intensive glycemic control improved this condition [74].

#### 4.7.2. Thiazolidinediones

Thiazolidinediones are orally administered hypoglycemic agents, which can be used as a monotherapy or in use with other hypoglycemic agents. Extensive use of thiazolidinediones can improve the glycemic control by decreasing 1% of HbA1C values [73]. The mechanism of action of thiazolidinediones in improving glycemic control and delaying the progression of DR via its antiangiogenic effect by inhibiting the activation of peroxisome proliferator-activated receptor-*γ* (PPAR-*γ*). PPAR-γ is a transcription factor that regulates the expression of genes located in the adipose tissue and retina [75].

#### 4.7.3. Biguanides

Biguanides, also known as metformin, is a proven hypoglycemic agent and has cardioprotective effects. It has been shown to process potent anti-inflammatory and antiangiogenic activities by decreasing the concentration of plasminogen activator inhibitor 1 and increasing the concentration of fibrinolytic activity [76, 77].

### 4.8. Hypolipidemic Agents

#### 4.8.1. Fibrates

Fenofibrate is used to treat hyperlipidemia by mainly lowering the triglyceride levels, total and low-density lipoprotein (LDL) cholesterol, small LDL cholesterol particles, and apolipoprotein B while increasing high-density lipoprotein (HDL) cholesterol. The results obtained from the FIELD study suggested that fenofibrate is a potential adjunct to photocoagulation. Besides, the effect of fenofibrate also acts via nonlipidemic mechanisms, mainly activating PPAR-*α* for DR prevention. Activation of PPAR-*α* mediated by fenofibrate led to inhibition of VEGFR2 expression and neovascularization in human umbilical endothelial cells. Moreover, fenofibrate also activates PPAR*α* to induce the expression of antioxidant enzymes, such as superoxide dismutase and glutathione peroxidase, and kills human umbilical endothelial cells via apoptosis induction. Further, the activation of PPAR-*α* has been shown to have protective effects on neuron cells [78–80].

#### 4.8.2. Statins

Treatment of atorvastatin alone has been shown to be ineffective in reducing DR, but it can reduce the need of laser treatment when use in combination as reported by Collaborative Atorvastatin Diabetes Study (CARDS) [81]. Besides, the contemporary therapy between fenofibrate and atorvastatin was carried by ACCORD-Eye sub study by using a modified Early Treatment DR Study (ETDRS) severity scale or laser photocoagulation for 4 weeks. The results showed 40% reduction in the relative risk of DR progression compared with placebo. The reduction of DR progression was due to a significant decrease in triglyceride levels and an increase in HDL levels [81, 82].

### 4.9. Antihypertensive Agents

#### 4.9.1. Angiotensin-2 Converting Enzyme Inhibitors

Blood pressure was reported as one of the contributing factors for the development of DR, as proper control of blood pressure reduced the development of DR in both type 1 and 2 diabetic patients. The most common drug used to treat hypertension in diabetic patients is angiotensin-2 converting enzyme (ACE) inhibitors because the renin-angiotensin system is also expressed in diabetic retinal [82, 83]. Recently, RAS study was carried out to evaluate the effect of blocking renin-angiotensin system using either ACE inhibitor (enalapril) or angiotensin receptor blocker (ARB) (losartan) on DR progression by examining renal and retinal morphologic characteristics in normotensive type 1 diabetics [84]. About 65% and 70% reduction in the rate of DR progression was seen after treatment with enalapril and losartan, respectively [84].

#### 4.9.2. Angiotensin-2 Receptor Blockers

Candesartan, an ATI-receptor blocker, was used in the Diabetic Retinopathy Candesartan Trial (DIRECT) to evaluate its protective effects against DR progression. This study was divided into three randomized double-blind placebo-controlled parallel group studies, with DIRECT-Prevent 1 represented a primary prevention study in type 1 diabetic patients without DR, DIRECT-Protect1 involving type 1 diabetic patients with DR, and lastly DIRECT-Protect2 was secondary prevention study that involves type 2 diabetic patients with DR. DIRECT-Prevent1 and DIRECT-Protect1 revealed that candesartan cannot prevent DR progression while no significant reduction in the progression of DR was found in DIRECT-Protect2. Despite this fact, candesartan could treat patients with mild DR [85].

#### 4.9.3. Antiplatelet Agents

Platelet activation is one of the side effects of chronic hyperglycemia in addition to retinal inflammation, aggregation, and thromboxane A2 accumulation. Aspirin is an antiplatelet agent and has been tested its effect on the progression of DR in EDTRS study. This study showed that aspirin treatment did not have any effects on disease progression or on the rates of vitreous hemorrhage. In addition, the possible effect of aspirin in slowing the progression of DR was observed when it used with dipyridamole [86, 87]. Moreover, Dipyridamole, Aspirin, Microangiopathy of Diabetes (DAMAD) study further showed that combination use of aspirin and dipyridamole caused a significant reduction in microaneurysms formation [86].

### 4.10. Other Potential Systemic Agents

#### 4.10.1. Ruboxistaurin

Ruboxistaurin (Arxxant®, Eli Lilly and Company) is a new class of compounds that is considered an investigational agent to treat NPDR. It has been shown to treat moderate to severe NPDR by reducing the overactivation of protein kinase C beta, which is involved in the pathogenesis of DR [88, 89]. Besides, it was reported to reduce the progression of DME and rates of vision loss in patients with DME [90, 91].

#### 4.10.2. Somatostatin Derivatives

Somatostatin possesses antiangiogenic activity in addition to its function as endogenous growth hormone inhibitor. Octreotide (Sandostatin®, Novartis) is an analog of somatostatin, which has been used as an anticancer drug for acromegaly, carcinoid tumors, and vasoactive intestinal peptide tumors. Treatment of octreotide decreased the rates of progression to PDR, vitreous hemorrhage, and the need for vitrectomy in patients with severe NPDR [92, 93].

#### 4.10.3. Potential Plant-Based Drugs

Plant-based drugs have also shown promising results in treating DR. The reasons of using plant-based drugs to combat DR disease are due to their safety, ability to produce hypoglycemic effects, and retinoprotective effect. One of the biochemical pathways that leads to DR induction is polyol pathway activation. This pathway metabolizes excess glucose in diabetes. The main enzyme in this pathway is aldoreductase (AR), which functions to reduce glucose in the retina to sorbitol using nicotinamide adenine dinucleotide phosphate (NADPH) as a cofactor [94, 95]. Since NADPH is consumed in this pathway, leading to lower antioxidant activity in retinal cells as NADPH is also required to act as a cofactor to produce intracellular glutathione. Decreased reduced glutathione leads to limited protective against oxidative stress. Generated sorbitol is impermeable to cellular membrane; thus, it accumulates in the cells before converting to fructose via slow metabolism [94, 95]. The remaining unconverted sorbitol has multiple damages to retinal cells, including osmotic damage [94, 95]. A few studies demonstrated the important role of polyol pathway in the pathogenesis of DR, in which AR inhibitors significantly reduced the incidence and severity of diabetic retinal lesions in galactose-fed animals [96–98]. During the activation of polyol pathway leading to DR, retinal ganglionic and endothelial cells are prone to damage because they are endowed with AR enzyme [6].

There are many AR inhibitors derived from plants, such as *Ocimum sanctum*, *Tinospora cordifolia*, *Azardiracta indica*, *Ganoderma lucidum*, and so forth *Ocimum sanctum* exerts its protective effect against DR when in use with vitamin E [99]. *Tinospora cordifolia* protects against DR by preventing retinal oxidative stress mediated by overexpression of proangiogenic and proinflammatory mediators [100]. Ganoderma lucidum has a protective effect on the retina from oxidative damage [101].

One of the plant-derived compounds is curcumin, which has been reported to prevent DR progression in a rat model by inhibiting the upregulation of retinal VEGF levels [102]. Further, curcumin was able to prevent the basement membrane from becoming thickened via antioxidant and anti-inflammatory mechanisms in the retina of treated rat [103]. Pycnogenol® is an extract from the bark of the maritime pine tree. This extract has potent antioxidant and anti-inflammatory activities, resulting in its use in the management of DR [103, 104]. Besides, chronic oral administration of genistein has been shown to prevent retinal vascular leakage in a rat model, possibly via the good tyrosine kinase inhibitory activity [105]. Green tea has also shown the good effect on DR, in which drinking green tea have proved to reduce the onset and incidence of DR [106, 107]. Hesperetin has also shown to have a preventive effect against DR [108]. Other compounds, such as quercetin and rosmarinic acid was showed to reduce DR by preventing angiogenesis via its antioxidant activity [109, 110].

#### 4.10.4. Possibility of Patient-Specific Photoreceptor Cell Replacement

Vision impairments are associated with the death of the light sensing photoreceptors cells of the outer neural retinal [111]. Transplantation-based photoreceptor cells replacement might be an attractive strategy to restore the retinal function, as the inner layers of the neural retinal of the majority of retinal degenerative patients connecting photoreceptor cells to the brain remained intact [112]. Moreover, the retinal does not have inhibitory myelin-associated proteins found in other central nervous system compartments [113].

A few cell types have been tested in the retinal degenerative models to restore the retinal functions. Cell types tested include retinal progenitor cells isolated from developing fetuses, and mature photoreceptor cells derived from post-mortem donor eyes [114–117]. Nonetheless, the post-mitotic photoreceptor precursor cells were found have the greatest capacity to survive, integrate with the remaining retinal cells to differentiate into mature photoreceptor cells upon post-transplantation [115, 118–122]. Further, recent advances in pluripotent stem cell technology enable researchers to generate photoreceptor precursor cells, which are difficult to obtain from human donor tissue due to the difference in cell differentiation and post-mortem degradation [111]. Under the controlled condition, functional photoreceptor precursors were able to derive from pluripotent stem cells and restored the retinal structure and function of the animal following transplantation [118, 120, 121, 123–125].

Recently, there are many debates about the suitability and clinical relevant of using either embryonic stem cell- (ESC-) and induced pluripotent stem cell- (iPSC-) derived retinal cells for transplantation [111]. The ethical concerns associated with ESC revolve around harvesting embryonic tissues for ESCs generation and immunological issue upon transplantation into unmatched recipients [126, 127]. It has been reported that patients transplanted with ESC-derived photoreceptor precursor cells require prolonged immunosuppressive therapy [128]. Nonetheless, ESCs can be generated into a sufficient quantity to treat many patients. Comparatively, the iPSC strategy allows the patients transplanted with genetically normal, immunological-match retinal cells when use with genome editing [129]. However, the iPSC method has its own drawbacks that autologous iPSC must be generated and validated per patient basis, resulting in this method being costly and requiring many skilled personnel per treated patient [111]. Interestingly, xeno-free and current Good Manufacturing Practice- (cGMP-) compatible iPSC protocols have been developed, which enhances the application of the iPSC method in nonprofit academic centers to treat regional patients [111].

Mesenchymal stem cells (MSCs) have been derived from bone marrow, cord blood, and peripheral blood as well as from other sources creates a high impact on cell therapy in eye diseases [130]. Adult bone marrow MSC which is positive for CD_90_ can partially differentiate into photoreceptors both in *in vivo* and *in vitro* and it is found to be neuroprotective in rat glaucoma model [131]. Adipose stromal cells (ASCs) have also gained increased attention because of its ability to preserve the transparency as well as it could get modified into functional keratocytes which suggests that it could be used as source for stromal regeneration in diseased corneas [132]. Intravitreal injection of ASC in streptozotocin- (STZ-) induced chronic diabetic model paired with host vasculature which suggests the possible pericyte replacement [133]. It was also shown to secrete Angiopoietin-1 (Ang-1) in a time-dependent manner, which is downregulated in DR especially when grown in culture medium containing vascular growth factors was found to promote reendothelialization [134]. It was also shown to improve blood glucose levels and maintain blood-retinal-barrier in STZ-induced DR rat model [135]. It was also shown to secrete several antiapoptotic, chemotactic, and anti-inflammatory proteins which were found to mediate beneficial effects of MSCs [136]. All these findings from rodents suggest that stem cells isolated from adipose tissue can be used as vehicle for the long term and sustained delivery of drugs to eye tissues and can replace the infected tissue with stem cells which might prove their beneficial effects [137]. Although the evidences are supportive to provide therapeutic approach for testing in human with retinal diseases, but further studies are warranted to specifically identify the role of these proteins in DR.

### 4.11. Antioxidants as Potent Therapeutic Agent

Partial restoration of diminished pericytes in retinal vessels is closely associated with administration of antioxidant treatment in diabetic rats [138]. During DR, oxidative stress has been closely linked to apoptosis of retinal cells and it leads to microvascular cell loss [139].

The antioxidant pharmacotherapy uses antioxidant enzymes, drug formulations, biogenic elements, and compounds with antioxidant activity [140]. The treatment criteria involving both intake of antioxidants through diet and supplementation. This procedure involves careful analysis of data and dosage of administration necessary to alleviate complications of diabetes and its side effects. N-acetylcysteine (NAC), vitamin C, and *α*-lipoic acid has been found to be effective in reducing diabetic complications. These may be administered through dietary intake or through ingestion of natural antioxidants both of which might be effective in therapy [141–143].

Clinical studies carried out in type I diabetic patients showed that high dose of vitamin E was found to restore blood flow in retina and prevent the loss of pericytes in diabetic rats through lipid peroxidation of the membrane [144, 145]. Vitamin C causes reduction in retinal neovascularization through suppression of superoxide production [146].

Calcium dobesilate has showed to decrease permeability of retina and reduce the expression of VEGF in diabetic rats. Although it has been widely used in many countries, the exact mechanism of action needs to be explored [147, 148]. Caffeic acid is an antiangiogenic agent which suppresses ROS production and VEGF expression in cells of the retina [149]. Lipoic acid attenuates apoptosis of rat retinal cells and prevents increase in nitrotyrosine levels and NF-*κ*B activation. It is also found to decrease the levels of VEGF and oxidation of proteins in retinal cells [150]. Rosmarinic acid significantly inhibits the retinal cell proliferation and showed to be nontoxic [151]. Benfotiamine, pyridoxamine, nicanartine, zinc, selenium, pycnogenol, curcumin, taurine, and green tea have been found to have various free radical scavenging properties and has been useful in treatment of DR. Benfotiamine, inhibits MnSOD, via blocking of the pathways involved in hyperglycemia [152]. Nicanartine, is antihypercholesterolemic which inhibits pericyte loss [153]. Zinc improved the levels of GSH in DR rats [154]. Selenium downregulates VEGF production in diabetic rats [155]. Taurine when supplemented long with selenium has been found to reduce biochemical alterations in diabetic rats [156]. Green tea polyphenols have great antioxidant potency which has been reported to improve SOD and GSH levels and reduce glucose levels [157]. Apart from administration or supplementation of individual antioxidants, a multiantioxidant administration might be the best therapeutic approach to treat DR [158].

Clinical trials involving compounds with the efficacy to inhibit protein kinase C and minimize oxidative stress which when used in combination with standard one might pave the way to successfully treat DR [159]. However, the dosage or concentration which was found to be effective in treating DR in animals is not enough to create beneficial impact on humans. Such discrepancies are not clear yet, but further studies are needed to determine the appropriate regimen [160].

The major downside in every disease condition is to maintain an effective drug concentration at the disease site for appropriate period to achieve expected pharmaceutical response. In such cases, use of polymeric adhesive nanoparticles may enhance the efficacy of drugs. Increasing drug retention time, slowing drug delivery, and high target specificity are the best therapeutic approaches for DR. Thus, an alternative for treating ocular pathology should provide cost-effective treatment. Combinatorial use of these antioxidants with nanoparticles might throw a limelight in this devastating area.

## 5. Conclusion

In the current scenario, the focus of the pharmacotherapy in combating the development and progression of DR is still mainly focused on corticosteroid therapy and anti-VEGF agents. What is the efficacy of compounds derived from plants? In this regard, further discovery of plant-based agents is needed. In addition, there have been some advances in the drug delivery devices for delivering drugs/proteins; there are still challenges to be overcome with regard to the particulate systems. For the long-term success of DR therapeutics, research options should consider considering the developing of drug delivery systems to have an extended period of effect for drugs/proteins, or further investigations on the possibility of using pluripotent stem cell technology for transplantation into DR host to restore retinal functions is needed.
