# Corrigendum to “Current Advances in Pharmacotherapy and Technology for Diabetic Retinopathy: A Systematic Review”

**DOI:** 10.1155/2018/5047142

**Published:** 2018-12-02

**Authors:** Lei Lu, Ying Jiang, Ravindran Jaganathan, Yanli Hao

**Affiliations:** ^1^Department of Ophthalmology, Yan'an People's Hospital, Qilipu St., Baota Qu, Yan'an, Shaanxi 716000, China; ^2^Pathology Unit, Preclinical Department, Faculty of Medicine, Royal College of Medicine Perak, Universiti Kuala Lumpur, No. 3, Jalan Greentown, Ipoh 30450, Perak Darul Ridzuan, Malaysia; ^3^Department of Ophthalmology, Yan'an University Affiliated Hospital, Yan'an, Shaan'xi Province 716000, China

In the article titled “*Current Advances in Pharmacotherapy and Technology for Diabetic Retinopathy*: *A Systematic Review*” [1], there was an error in the second and third affiliations. The corrected affiliations are shown above.

In the “4.2. Corticosteroid Therapy with Sustained Delivery System” subsection, the following should be updated:

1. The text reading “It has been reported by a FAME study that low dose of Iluvien inserted (0.19 mg per day) showed improved BCVA compared to higher dose [14, 15, 39]. Currently, Iluvien is still under investigation by FDA to approve its application to the market [13]” should be corrected to “It has been reported by a FAME study that low dose of Iluvien inserted (0.2 mg per day) showed improved BCVA compared to higher dose [14, 15, 39]. At 3 years in the FAME trials, Iluvien (0.2 *µ*g fluocinolone acetonide per day) led to 28.7% of patients achieving a ≥15 letter score improvement in visual acuity versus 18.9% in the sham group. This effect was even greater in a preplanned analysis of the data assessing the impact in patients with persistent or recurrent diabetic macular edema (DME) and in this group Iluvien led to 34.0% achieving a ≥15 letter score improvement versus 13.4% in the sham group. Based on the results of the FAME pivotal trials, Iluvien is now approved for use in DME in Europe and the USA.”
2. The sentence reading “In comparison, another fluocinolone acetonide intravitreal implant, Retisert® (Bausch and Lomb), is approved by FDA for the treatment of chronic, noninfectious uveitis. However, its treatment caused severe cataract and glaucoma in a phase III clinical trial [14, 15]” should be removed.
